# Synthesis and Characterization of a Conductive Polymer Blend Based on PEDOT:PSS and Its Electromagnetic Applications

**DOI:** 10.3390/polym14030393

**Published:** 2022-01-19

**Authors:** Hong-Kyu Jang, Jinbong Kim, Ji-Sang Park, Jin Bum Moon, Jaecheol Oh, Woo-Kyoung Lee, Min-Gyu Kang

**Affiliations:** Composites Research Division, Korea Institute of Materials Science, 797, Changwon-daero, Seongsan-gu, Changwon-si 51508, Korea; jbkim@kims.re.kr (J.K.); jspark@kims.re.kr (J.-S.P.); jbmoon@kims.re.kr (J.B.M.); jcoh@kims.re.kr (J.O.); hayon@kims.re.kr (W.-K.L.); medsgn@kims.re.kr (M.-G.K.)

**Keywords:** conductive polymer blend, PEDOT:PSS, polyurethane, electrical conductivity, screen-printing, conductive thin film, electromagnetic wave absorber

## Abstract

The purpose of this study is to prepare a resistive lossy material using conducting polymers for electromagnetic wave absorbers. This paper presents a conductive paste largely composed of poly(3,4-ethylenedioxythiophene):poly(styrenesulfonate) with a polyurethane binder. The various secondary compounds are added in small amounts to an aqueous blended solution in order to enhance the electrical and mechanical properties of the conductive thin film. The synthesized conductive paste is characterized through electrical, chemical, and morphological analyses. The electrical conductivity of the thin film is measured using a four-point probe and surface profiler. The chemical and morphological changes are studied in various experiments using a Raman microscope, X-ray photoelectron spectroscopy, a scanning electron microscope, and an atomic force microscope. In order to verify the applicability of the synthesized conductive paste, which is composed of 70 wt% PEDOT:PSS, 30 wt% polyurethane, and secondary additives (DMAE 0.4 wt%, A-187 0.5 wt%, DMSO 7 wt%, Dynol 604 0.1 wt%, PUR 40 2.5 wt%), the Salisbury screen absorber is fabricated and evaluated in the X-band. According to the results, the absorber resonates at 9.7 GHz, the reflection loss is −38.6 dB, and the 90% absorption bandwidth is 3.4 GHz (8.2 to 11.6 GHz). Through this experiment, the applicability of the PEDOT:PSS-based conductive paste is sufficiently verified and it is found that excellent radar-absorbing performance can be realized.

## 1. Introduction

Conductive materials used in various technical and commercial applications are required to combine all properties necessary for commercialization, such as high electrical conductivity, good processability, and long-term stability. However, most conjugated polymers have also shown some disadvantages in the past when used for industrial applications: environmental vulnerability, thermal instability, low solubility, and poor processability [1,2,3]. According to previous studies, poly(3,4-ethylenedioxythiophene):poly(styrenesulfonate) or PEDOT:PSS is considered to be a promising candidate for creating conductive materials because it can satisfy the mentioned requirements, such as good film-forming properties, high conductivity, high transparency, and excellent stability [4,5]. The processing and film-forming properties of the PEDOT:PSS solution can be adjusted and improved by adding small amounts of secondary compounds, such as binders, solvents, surfactants, coupling agents, and thickening agents [6,7]. In addition, the electrical conductivity of the PEDOT:PSS thin films can be increased by treatment with high-boiling polar compounds, e.g., dimethylsulfoxide (DMSO), N,N-dimethylformamide (DMF), ethylene glycol (EG), and tetrahydrofuran (THF) [7,8,9].

Conventional radar absorbing materials (RAM), carbon-based dielectric materials, and ferrite-based magnetic materials have some inherent disadvantages. The nano-sized powder used in lossy materials increases their structural weight and degrades their mechanical properties. Moreover, it is difficult to control the electrical properties of composites, such as their permittivity, permeability, and conductivity, by the addition of filler content [10]. On the other hand, conducting polymers are considered to be promising alternative materials for electromagnetic (EM) wave absorbers due to their various advantages over other traditional materials, e.g., their light weight, ease of production, controllable conductivity, good processability, and broadband radar absorption [11,12,13,14]. Truong [11] developed RAM by combining poly(pyrrole) powder with paint and rubber. In particular, the performance change of RAM according to long-term temperature aging was evaluated, and the stability and applicability of the conducting polymer were evaluated. Wong [12] used poly(pyrrole) to design a Salisbury screen absorber. Their study was the first case that revealed the electromagnetic absorbing characteristics through analysis and experiments by applying a conducting polymer to a large area. Franchitto [13] mixed poly(aniline) with rubber to develop a 3.0 mm thick RAM with −10 dB bandwidth at 9.4–12.4 GHz within the X-band. Finally, Wright [14] used poly(aniline) to manufacture a Salisbury screen absorber and, through analysis, the radar-absorbing performance was observed to change with the adjustment of the material electrical conductivity, absorber thickness, and capacitance.

PEDOT:PSS-based conductive materials are widely used for various applications, such as in sensors, batteries, memory, solar cells, and electronic circuits, due to their unique mechanical and electrical properties. In particular, this material can be applied to develop RAM for use in the field of stealth technology to make the military purpose systems less visible to detection radar [10,15]. The main purpose of this study was to prepare a resistive lossy material using conducting polymers for electromagnetic wave absorbers. Herein, we present a conductive paste largely composed of PEDOT:PSS with a polyurethane binder. The synthesized conductive paste was examined and characterized through electrical, chemical, and morphological analyses. To demonstrate the applicability of the conductive paste for EM wave absorbers, a Salisbury screen absorber with a target frequency of 10.0 GHz was fabricated and its radar-absorbing performance was evaluated in the X-band (8.2 to 12.4 GHz).

## 2. Synthesis

### 2.1. Materials for Conductive Paste

Despite the various advantages offered by conducting polymers, they have been used in limited regions as the material is difficult to process in a suitable form for applications because of its low solubility and mechanical strength. In order to overcome such disadvantages, various methods have been proposed, such as altering the monomer structure, synthesizing a precursor with solubility, and applying solubility using a special dopant. Additionally, the weak mechanical properties can also be improved by mixing or hybridizing the conducting polymer with general-purpose binders [6]. In addition, the electrochemical properties of the conducting polymer can be controlled or improved through synthesis with various organic solvents [8,9,16].

In this research, we synthesized a conductive polymer blend with suitable electrical and mechanical properties for use in an electromagnetic wave absorber. The conducting material was an aqueous PEDOT:PSS solution, Clevios PH 500 (Heraeus Co., Ltd., Hanau, Germany), with high electrical conductivity and superior thermal and electrochemical stabilities. The binder material was a water-soluble polyurethane, NPC-3600 (Nanux Co., Ltd., Changwon, Korea), with outstanding wear resistance and solvent resistance. In the case of the main materials, Clevios PH 500 and NPC-3600, there was a large difference in acidity, with one being acidic and one alkaline. Therefore, if they were mixed directly, the mixture would be chemically unstable and the problem of crystals forming in the mixture would occur. In order to solve these problems, the chemical stability of the mixture was secured by controlling the acidity of Clevios PH 500 through the addition of a small amount of the hyper-alkaline solvent dimethylaminoethanol (DMAE), diluted in deionized water.

To increase the processability of the conductive paste through a simple printing process such as screen printing, the mechanical and chemical properties of the conductive paste were improved by adding a small amount of coupling agent, Silquest A-187 (Momentive Performance Materials Inc., Waterford, NY, USA), and surfactant, Dynol 604 (Evonik Industries Co., Ltd., Essen, Germany) [7,17]. One of the greatest advantages and characteristics of the PEDOT:PSS conducting polymer is that the electrical conductivity can be increased from tens to hundreds of times greater through synthesis with organic solvents. A representative conductivity enhancement additive is the amide N-Methylpyrrolidone (NMP), dimethylformamide (DMF), and the dimethylsulfoxide (DMSO). According to various studies, DMSO is widely used and can effectively increase the electrical conductivity of PEDOT:PSS polymer film [9,16,18]. In addition, to improve the workability and surface quality of the conductive thin film fabricated by the screen-printing process, a small amount of rheology modifier was added to adjust the viscosity of the conductive paste to fit the screen-printing process. The rheology modifier used was Tafigel PUR 40 (Münzing Chemie Co., Ltd., Abstatt, Germany) and we took into consideration the polyurethane binder material. The following Table 1 summarizes the information of the conductive material, the binder material, and the various secondary compounds to synthesize the conductive paste.

### 2.2. Conductive Polymer Blend Based on PEDOT:PSS–Polyurethane

**Figure 1:**
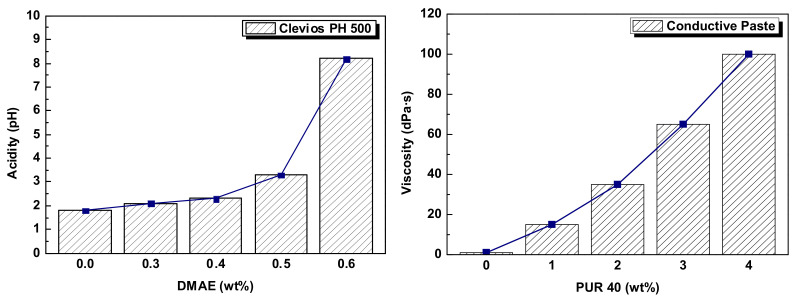
Change in acidity of Clevios PH 500 and the viscosity of conductive paste.

In the fabrication of a conductive paste, the stability, processability, and workability of the paste can be improved through synthesis with various organic solvents, and its electrical and mechanical characteristics can be enhanced as well. Therefore, it is very important to select an appropriate organic solvent and determine the amount of addition. In this research, in order to prevent any chemical instability of the mixture that could occur due to the difference in acidity between the main materials, DMAE solution diluted to 50% in deionized water was added to investigate the acidity change of the mixture. The acidities, measured by a portable HM-30P pH meter (TOA-DKK Co., Ltd., Tokyo, Japan), were pH 1.8 for Clevios PH 500, pH 8.0 for NPC-3600, and pH 12.0 for the 50% DMAE diluent. The 50% DMAE diluent was added in an amount of 0.3–0.6 wt% based on the Clevios PH 500 and mixed with a magnetic stirrer for 10 min at a rotational speed of 500 rpm. As shown in Figure 1, with the increase in the amount added, the acidity gradually increased from pH 1.8 (0.3 wt%) to pH 3.3 (0.5 wt%) and then rapidly increased to pH 8.2 at 0.6 wt%. In particular, because the electrical property of Clevios PH 500 is related to the acidity of the solution, the appropriate control of the amount of DMAE solution added is necessary to keep the acidity of the final synthesized conductive paste below pH 8.0. According to the final experimental result, after adding 0.4 wt% of DMAE diluent, the mixed solution of Clevios PH 500 and NPC-3600 showed an acidity of PH 6.5.

The binder material NPC-300 polyurethane was mixed with Clevios PH 500 for 15 min using an agitator with a rotational speed of 600 rpm to improve the mechanical and physical properties of the conductive paste. Here, the mixing ratio was determined based on the electrical conductivity required by the conductive paste. In this paper, a representative case of mixing the binder material at 30 wt% based on the conductive material is described. The detailed process of synthesis with secondary additives to enhance and improve the electrical and physical properties of the conductive paste is described. To increase the binding force of the paste and its adhesiveness to the target surface, the coupling agent Silquest A-187 was added at a volume of 0.5 wt% based on the mixture (Clevios PH 500 + NPC-3600) for 15 min at the rotational speed of 6000 rpm.

**Figure 2:**
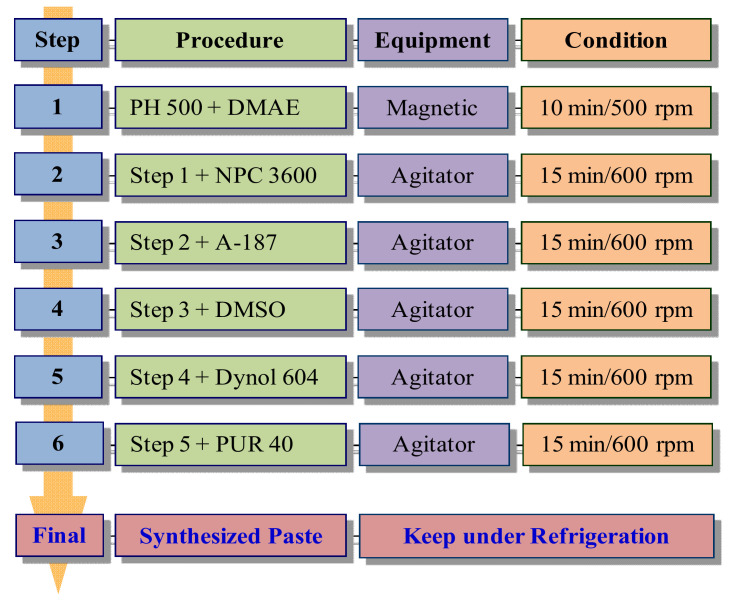
Synthesis formulation of conductive paste.

Additionally, to improve the electrical conductivity, DMSO was added in an appropriate amount. Generally, the addition of DMSO results in a rapid increase in the solution’s electrical conductivity with an increase in the addition amount. However, when the added amount is continuously increased, the rate of increase is gradually slowed down until saturation is reached; then, the electrical conductivity rapidly decreases [18,19]. Thus, the added amount has to be below 15 wt% based on the solution. Next, in order to enhance the surface-coating properties in the thin-film fabrication, the surfactant Dynol 604 was added at a volume of 0.1 wt% based on the solution and then mixed under the same conditions. Lastly, before controlling the viscosity of the conductive paste to fit the screen-printing process, a polypropylene filter bag (BPONG-10P3P, Filter Specialists Inc., Michigan, IN, USA) was used for filtering; then, we added the rheology modifier PUR 40 at 2–3 wt% based on the solution to control the viscosity of the paste. Figure 2 schematically shows the mixing sequence and mixing method of the PEDOT:PSS conducting polymer, the polyurethane binder, and the various secondary additives to synthesize the conductive paste.

## 3. Characterization

### 3.1. Electrical Analysis of Conductive Paste

**Figure 3:**
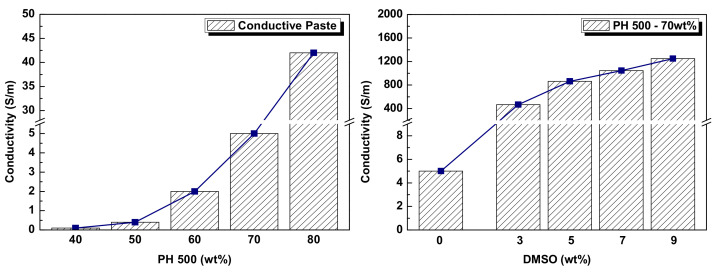
Conductivity of conductive paste measured by Clevios PH 500 and DMS.

The electrical conductivity of the synthesized paste was determined based on the ratio between conducting and non-conducting materials, PEDOT:PSS and polyurethane. In this study, the conductive pastes were mixed in proportions of 40, 50, 60, 70, and 80 wt% by weight of Clevios PH 500 to blended solution (PH 500 + NPC-3600). To measure the electrical conductivity of each paste, conductive thin films were printed on a soda lime glass plate (100 × 100 × 0.7 mm) by a fabrication process using a bar-coating device (KP-300, Kipae E&T Co., Ltd., Hwaseong, Korea). To calculate the electrical conductivity of the synthesized paste, a surface profiler (XP-1, Ambios Technology Inc., Santa Cruz, CA, USA) and 4-point probe (SR2000N, AIT Co., Ltd., Suwon, Korea) were used to measure the coating thickness and surface resistance of the fabricated specimen. According to the results on the left-hand side of Figure 3, the electrical conductivity of the thin films increased from 0.1 to 42 S/m as the PEDOT:PSS content increased.

The most significant characteristic of the PEDOT:PSS conducting polymer is that the electrical conductivity of the PEDOT:PSS films can be enhanced by more than tens or hundreds of times by the addition of polar solvents to the PEDOT:PSS solution. Several studies have reported that DMSO solvent is the best material for effectively increasing the electrical conductivity [6,12]. In this study, pastes composed of 70 wt% Clevios PH 500 and 30 wt% NPC-3600 were prepared with different concentrations by weight of DMSO to show the conductivity increase. The mixing ratios of DMSO and blended solution (PH 500 + NPC-3600) were 0, 3, 5, 7, and 9 wt% by weight of DMSO, respectively [19,20]. According to the results shown on the right-hand side of Figure 3, the thin-film conductivity of DMSO paste added at an amount of 3 wt% rapidly increased by 93 times from 5 to 465 S/m compared with the pristine paste. In addition, although the rate of increase was gradually decreased, the conductivity increment was a function of DMSO concentration up to 9 wt%, which corresponded to the maximum value of 1249 S/m.

### 3.2. Chemical Analysis of Conductive Paste

The conductivity enhancement of the PEDOT:PSS film by the addition of high-boiling solvents and polar compounds such as DMSO, DMF, THF, and EG is well known and has been widely studied. Despite the progress in the electrical conductivity of PEDOT:PSS, the mechanism behind these conductivity enhancements is debatable [8]. Huang [9] and Rwei [21] proposed that structural changes may cause the conductivity improvement. The PEDOT polymer structure changed from a coil-shaped form to a linear and expanded coil-shaped form due to the secondary additive, and this change improved the mobility of the charge carrier within the chain or between chains, leading to an improvement in the electrical conductivity. Na [20] proposed morphological changes to be the reason. This is because the size of the PEDOT:PSS polymer particle increases and the PSS part with electric insulation becomes thinner, meaning that the surface area of the relatively conductive material PEDOT increases, making the movement of charge easier and increasing the electrical conductivity. On the other hand, Wang [22] proposed that the conductivity increased due to the decrease in the PEDOT:PSS particle size and the even distribution based on SEM results. In this paper, we study how the electrical conductivity is increased by the addition of DMSO through qualitative and quantitative analysis of the conductive paste comprising 70 wt% Clevios PH 500 and 30 wt% NPC-3600.

**Figure 4:**
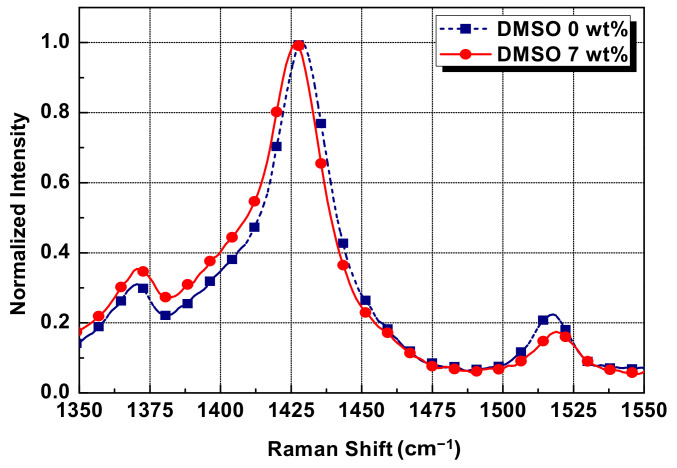
Raman spectra of pristine film and film with 7 wt% DMSO paste.

To understand the mechanism of the conductivity enhancement, the films made of pristine paste and DMSO-treated paste were analyzed using a Raman microscope (LabRAM HR UV-VIS-NIR, Horiba Jobin Yvon Co., Ltd., Kyoto, Japan). Raman spectroscopy is an effective method for studying the chemical structure of conducting polymers. The Raman spectra of the DMSO-untreated and -treated films are shown in Figure 4. The main difference between the two pastes is the Raman shift; the pristine film exhibited a peak at 1446 cm^−1^, whereas the film with the addition of DMSO exhibited a slightly left-shifted peak at 1449 cm^−1^. The Raman frequency is dependent on the covalent bond strength and the atomic mass, and the Raman spectrum represents how much the frequency of the scattered light has changed in relation to the Rayleigh scattering.

The general PEDOT molecular structure is a benzoid and quinoid structure and these two structural forms coexist. The benzoid structure has two C=C covalent bonds in the tiophene with a coil shape, while the quinoid structure has one C=C covalent bond in the tiophene with a linear and expanded coil form. The Raman frequency in the 1400–1500 cm^−1^ band of the PEDOT Raman spectrum is related to the C=C covalent bond vibration. In other words, the PEDOT molecular structure chains are partially changed from a benzoid structure to a quinoid structure with relatively weak bond strength by the addition of DMSO, resulting in the Raman spectrum moving to the left. Consequently, as the PEDOT polymer structure changes to a linear or expanded coil shape, the movement of the charge carrier within or between the chains of the neighboring PEDOT polymers is enhanced and the electrical conductivity is improved [23,24].

**Figure 5:**
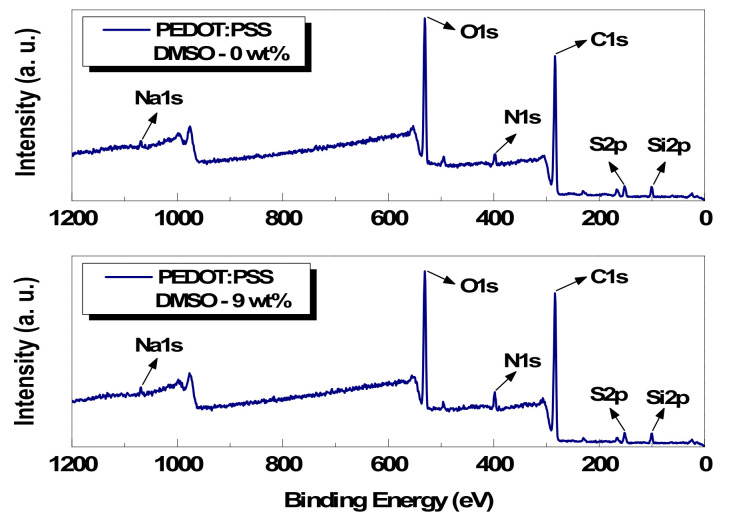
Wide-scan survey spectra of pristine and 9 wt% DMSO paste film.

X-ray photoelectron spectroscopy (XPS) is a quantitative analysis technique used to investigate the chemistry at the surface of a material. In this paper, DMSO-untreated and -treated paste films were analyzed to observe the composition and composing element change in the conductive film surface using XPS (Sigma Probe, Thermo Fisher Scientific Inc., Waltham, MA, USA). Figure 5 shows the analysis graph of the composing elements based on the binding energies of each peak, made using the Thermo Advantage program from the measured signal. Representatively, examining the oxygen (O), carbon (C), and sulfur (S) atoms, the peak location of oxygen O(1s) is 531 eV, that of carbon C(1s) is 284 eV, and that of sulfur S(2P) is 152 eV. Based on the composing element analysis results, a quantitative analysis of the DMSO-untreated and -treated conductive paste films was performed. In this study, the quantitative analysis experiment was conducted on carbon and sulfur atoms.

**Figure 6:**
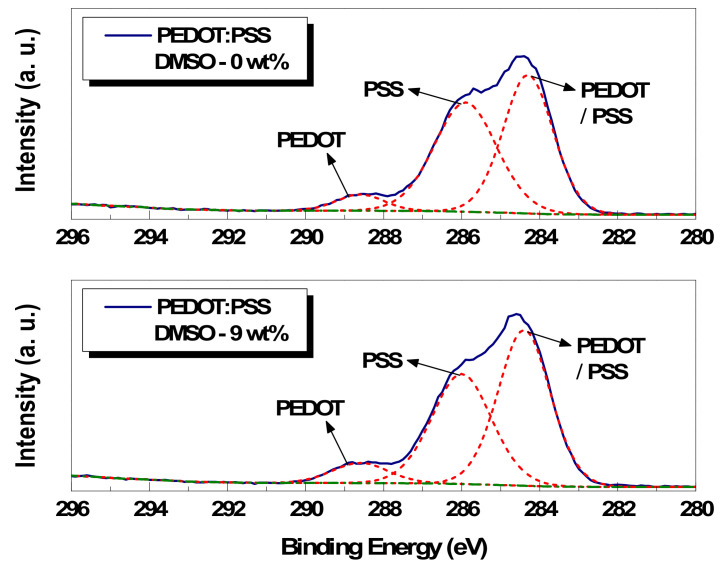
C(1s) core-level spectra of pristine and 9 wt% DMSO paste film.

First, the peak fitting analysis results on the carbon atom C(1s) core-level spectra of pristine and 9 wt% DMSO-treated paste film are shown in the graph of Figure 6 as a representative case. In addition, the relative area ratios of each component for the 0, 3, 5, and 9 wt% DMSO-treated conductive pastes are organized in Table 2, respectively. The carbon element can be separated into three peaks, where the highest binding energy is due to the carbon atom bonded to the C–O–C structure of the PEDOT molecule and the middle binding energy is from the carbon atom linked to the SO_3_^−^ group of the PSS molecule. The lowest binding energy is due to the rest of the carbon atoms of the PEDOT molecule and PSS molecule [20]. The composition ratio changes in the surface before and after the addition of DMSO were found by analyzing the relative area ratio of each component. According to the comparison results, the area of the carbon atom linked to the SO_3_^−^ group of the PSS molecule was gradually reduced by 10.6%, 11.9%, and 11.7% as the amount of DMSO added increased (0, 3, 5, and 9 wt% of DMSO). In addition, the carbon atom area bonded to C–O–C of the PEDOT molecule was gradually increased by 13.2%, 18.9%, and 34%, respectively. These results signify the change in the composition ratio of the PEDOT and PSS molecules on the specimen surface and the reduction in the PSS molecule ratio.

**Figure 7:**
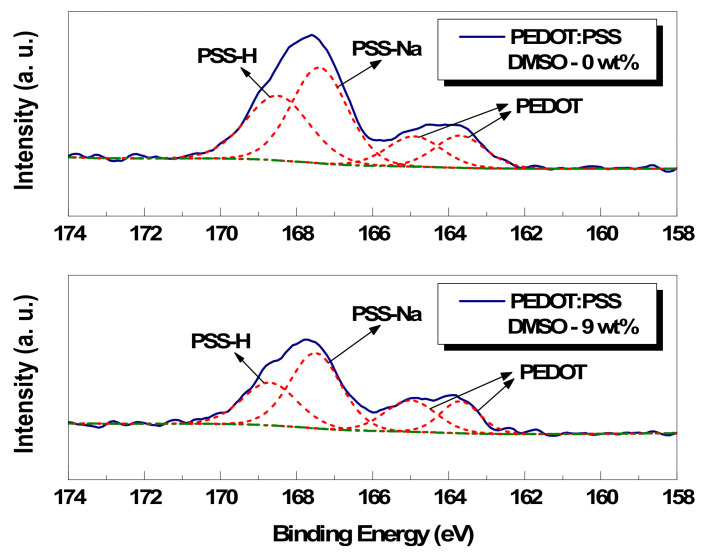
S(2p) core-level spectra of pristine and 9 wt% DMSO paste film.

Second, the peak fitting analysis results on the sulfur S(2P) core-level spectra of pristine and 9 wt% DMSO-treated paste film are shown in the graph of Figure 7 as a representative case. In addition, the relative area ratios of each component for the 0, 3, 5, and 9 wt% DMSO-treated conductive pastes are organized in Table 3, respectively. The sulfur atoms can be separated into four peaks, where the two peaks in the low-binding-energy range are due to the PEDOT molecule and the two peaks in the high-binding-energy range are due to the PSS molecule. Analyzing each peak in more detail, it can be seen that the PEDOT molecule is composed of the neutral-state PEDOT (163.7 eV) and the ionized-state PEDOT^+^ (164.9eV). Additionally, the PSS molecule is composed of PSS^−^-Na^+^ (167.4 eV) and PSS^−^-H^+^ (168.5 eV) [25,26]. By comparing the PEDOT and PSS molecule area ratios at the surface before and after the addition of DMSO, it can be seen that the ratio of 1:2.9 before the addition changed to 1:2.8, 1:2.4, and 1:2.2 after the addition of 3, 5, and 9 wt% of DMSO, respectively. These results signify the change in the composition ratio of the PEDOT and PSS molecules on the specimen surface and the reduction in the PSS molecule ratio.

### 3.3. Morphological Analysis of Conductive Paste

**Figure 8:**
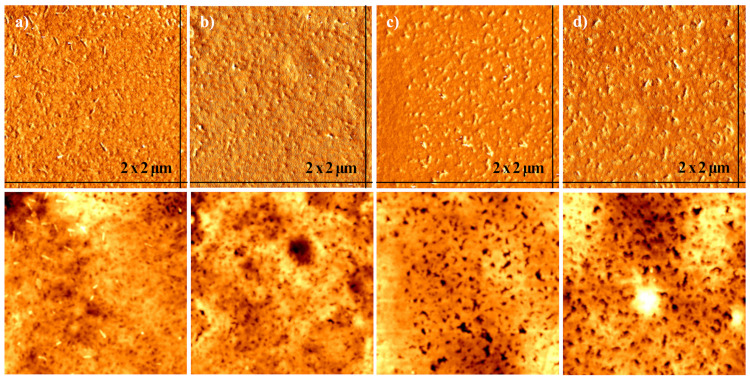
AFM images of (**a**) 0 wt%, (**b**) 3 wt%, (**c**) 5 wt%, and (**d**) 9 wt% DMSO paste film.

The morphological analysis of the film fabricated with conductive paste before and after treatment with DMSO solvent was conducted using microscopy analysis techniques to support the chemical analysis results. The morphological change at the specimen surface was tested using an atomic force microscope: AFM XE-100 (Park System Co., Ltd., Suwon, Korea) (AFM). Figure 8 shows the phase and topographic images of the 2 × 2 µm^2^ specimen surface obtained using AFM. The experiment was conducted on the thin films printed by using a conductive paste with DMSO solvent added in amounts of 0, 3, 5, and 9 wt%. According to the analysis results, as the amount of DMSO increases, the particle size and the roughness of the surface also increases. In other words, as the DMSO organic solvent is added, the composition ratio of the surface is changed and the amount of insulating PSS component decreases, while the conductive PEDOT component continues to increase so that the PEDOT particles become relatively large and the surface becomes rough. As a result, since the PEDOT particle size increases and the insulating PSS becomes thinner, the electrical conductivity of the surface increases [27,28]. These morphological analysis results are in good agreement with the XPS analysis results.

**Figure 9:**
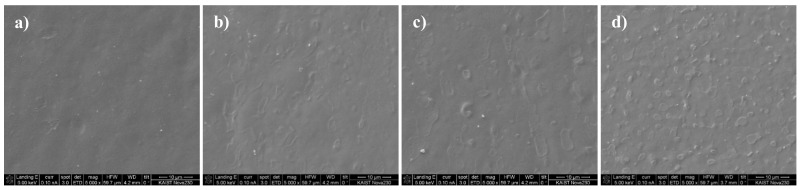
SEM images of (**a**) 0 wt%, (**b**) 3 wt%, (**c**) 5 wt%, and (**d**) 9 wt% DMSO paste film.

In addition, the morphological analysis was conducted using a scanning electron microscope. Figure 9 shows the SEM images at a × 5000 magnification of the thin films printed by using a conductive paste with DMSO solvent added in amounts of 0, 3, 5, and 9 wt%. According to the results, as the amount of solvent increases, the size of the PEDOT:PSS polymer agglomeration decreases and the overall distribution becomes uniform. Consequently, the conductive PEDOT particles are uniformly distributed and the connection between the chains of the neighboring PEDOT polymers is enhanced so that the electrical conductivity increases [9,16,22]. These morphological analysis results are in good agreement with the chemical analysis results, which showed that the PEDOT polymer structure changes to a linear or expanded coil shape.

## 4. Applications

### 4.1. Design of EM Wave Absorber

**Figure 10:**
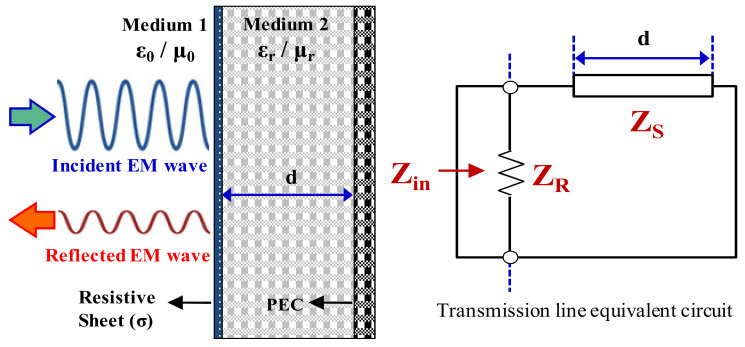
Salisbury screen absorber and transmission line model.

In this paper, the additional verification of the EM wave-absorbing capability is necessary in order to determine the applicability of the synthesized conductive paste in a radar shielding and absorber. The most representative form among the various screen-type radar absorbers that feature a conductive thin film is the Salisbury screen absorber. The Salisbury screen absorber has a simple structure made up of a resistive sheet, dielectric substrate, and metal ground layer, as shown on the left-hand side of Figure 10. When the electromagnetic wave propagates through the free space to reach normal incidence with a Salisbury screen absorber, it can be expressed as a transmission line model using equivalent impedance, as shown on the right-hand side of Figure 10. The condition for no reflection of the incident electromagnetic wave is when the input impedance (Z_in_) of the Salisbury screen absorber is equal to the impedance of the free space (i.e., air). The input impedance of the Salisbury screen is the resistive sheet impedance (Z_R_)_._ If the resistive sheet is 377 Ω/sq (the impedance of air), then good impedance matching to minimize reflections is achieved [29].

**Figure 11:**
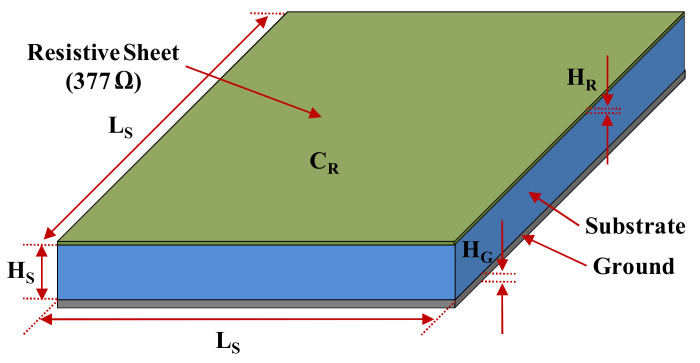
Design of the Salisbury screen absorber.

The Salisbury screen absorber was designed and fabricated to experimentally verify the applicability of the developed conductive paste to a radar absorber. The electromagnetic field analysis program CST Microwave Studio (CST-MWS) was used to design the radar absorber. The basic design properties were the conductivity of the material for resistive sheets and the permittivity of the glass fiber/epoxy composite for substrates. The resistive material used was synthesized conductive paste with a conductivity of 1000 S/m, and the dielectric substrate used was a plain-weave glass fiber/epoxy composite, where the permittivity was measured using a waveguide and a network analyzer. The resistive sheet thickness and dielectric layer thickness were calculated theoretically. The rest of the necessary values for the design are shown in Figure 11 and Table 4.

The reflection loss of the designed Salisbury screen absorber was simulated by using the CST-MWS software. According to the simulation results, resonance occurred at the design target frequency of 10.0 GHz, and the minimum reflection loss was −38.1 dB. Additionally, the 90% radar-absorbing bandwidth (−10 dB) within the X-band (8.2–12.4 GHz) was 3.8 GHz from 8.2 to 12.0 GHz.

### 4.2. Fabrication of EM Wave Absorber

The Salisbury screen absorber was manufactured with a resonance frequency of 10.0 GHz. The resistive sheet used for the Salisbury screen was the Clevios PH 500 at a content of 70 wt% conductive paste with an electrical conductivity of approximately 1000 S/m and GEP 118 plain-weave glass fiber/epoxy prepreg. For the dielectric substrate and conductive ground layer, we used GEP 118 plain-weave glass fiber cloth and WSN 3K plain-weave carbon fiber cloth (SK Chemical Co., Ltd., Seongnam, Korea), respectively. The epoxy resin and hardener (RIM 135, RIM 134, Hexion Inc., Columbus, OH, USA) were used as the binding materials.

**Figure 12:**
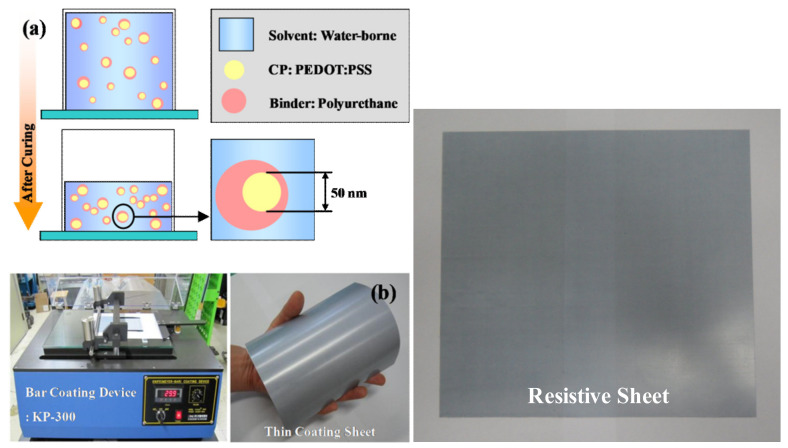
Fabrication of the thin resistive sheet: (**a**) thin film coating process (**b**) resistive sheet.

First, in order to fabricate a resistive sheet with a surface resistance of 377 Ω/sq, GEP 118 glass fiber/epoxy, the same as that used in the dielectric layer, was used to prepare a sheet of 150 µm thickness through the autoclave process. Next, to manufacture a resistive film with a thickness of 2.65 µm, the conductive paste was printed onto a prepared glass fiber/epoxy sheet using the KP-300 film-coating equipment and bar, and then cured for 30 min at 130 °C in a thermal oven. Based on the experimental results, the fabricated conductive thin film had a thickness ranging from 2.59 to 2.72 µm and an average thickness of 2.64 µm. Additionally, a portable sheet resistance meter (RC-2175, EDTM Inc., Toledo, OH, USA) was used to evaluate whether the sheet resistance of the fabricated resistive sheet was 377 Ω/sq. The measurement result showed that the sheet resistance ranged between 365 and 385 Ω/sq and that the average value was 379 Ω/sq. Figure 12 shows the detailed fabrication process and configuration of the resistive thin sheet.

**Figure 13:**
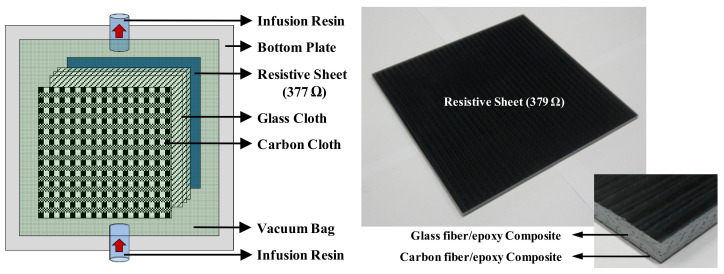
Fabrication of the Salisbury screen absorber.

In this study, in order to fabricate a Salisbury screen absorber composed of a resistive sheet, dielectric layer, and conductive ground layer, a radar absorber structure was manufactured using the infusion process. Figure 13 schematically shows the stacking sequence and fabrication process used to manufacture the Salisbury screen absorber through the infusion molding process. The manufacturing process can be divided largely into the material stacking stage and the resin infusion and curing stage. First, the prepared resistive film, glass fiber fabric, and carbon fiber fabric were laminated on the mold in order from the bottom. The GEP 118 glass fiber fabric had a thickness of 160 µm after curing. Thus, 22 plies had to be stacked in order to fabricate a 3.5 mm dielectric substrate for the Salisbury screen absorber. Next, at the resin infusion and curing stage, the mixed binding material at a ratio of 100:30 of epoxy to hardener was infused into the stacked materials using the vacuum pressure of a vacuum pump and cured at room temperature.

### 4.3. Electromagnetic Wave-Absorbing Performance

**Figure 14:**
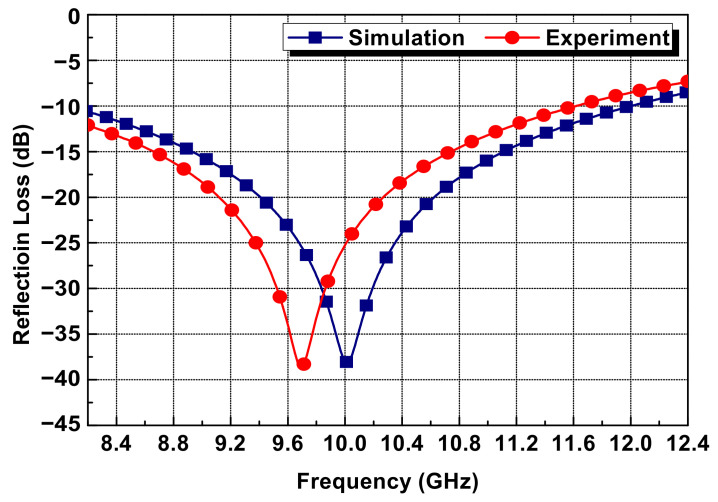
EM wave-absorbing performance of the Salisbury screen absorber.

The performance of the Salisbury screen absorber using the resistive thin film fabricated by the PEDOT:PSS–polyurethane-based conductive paste was determined. In order to evaluate the radar-absorbing performance, the free space measurement system (Microwave Measurement Systems, State College, PA, USA) was used to measure the reflection loss of the fabricated radar absorber at the X-band (8.2–12.4 GHz). To compare the reflection loss of the Salisbury screen absorber through simulations and experiments, the two results are shown together in the graph of Figure 14. According to the measurement results, resonance of the fabricated absorber occurred at the frequency of 9.7 GHz with a reflection loss of −38.6 dB. Additionally, the −10 dB absorbing bandwidth within the X-band was 3.4 GHz and the 90% radar absorption occurred at 8.2–11.6 GHz.

The cause of the change in the resonance frequency of the fabricated Salisbury screen absorber was largely manufacture error and measurement error. However, there is a probability that the error caused during the manufacturing process was greater than the measurement error. In actuality, the dielectric substrate produced by the RTM process had a thickness of 3.62 mm, which is 0.12 mm thicker than the design value of 3.5 mm. Therefore, the resonance frequency of the absorber shifted in the low-frequency direction. To verify this cause of the error, the CST-MWS program was used to simulate the performance of the radar absorber with a 3.62 mm substrate thickness again, which resulted in almost the same reflection loss graph as the measurement results.

## 5. Discussion and Conclusions

In this research, a study was conducted aiming to develop a resistive lossy material using a conducting polymer for application to an electromagnetic wave absorber. We synthesized the conductive paste using a PEDOT:PSS conductive polymer with high electrical conductivity and electrochemical stability, Clevios PH 500, and a water-soluble polyurethane binder with excellent mechanical properties, NPC-3600. In particular, many experiments were conducted and various databases were constructed using various secondary additives, such as PH control solvent, coupling agent, conductivity enhancer, surfactant, and thickening agent, to enhance and improve the mechanical and electrical properties. Considering both the performance and processability of the EM wave absorber using a low-cost screen-printing process, the conductive paste composed of 70 wt% Clevios PH 500, 30 wt% NPC-3600, and secondary additives (DMAE 0.4 wt%, A-187 0.5 wt%, DMSO 7 wt%, Dynol 604 0.1 wt%, PUR 40 2.5 wt%) was found to be the optimal compounding formulation.

In addition, a chemical analysis using a Raman microscope and X-ray photoelectron spectroscopy and morphological analyses using an atomic microscope and scanning electron microscope were performed to study the electrical conductivity improvement mechanism of the PEDOT:PSS-based conductive paste film according to the DMSO organic solvents. According to the qualitative and quantitative analyses, several causes of improvement in electrical conductivity can be identified. The first is to change the PEDOT molecular structure from a benzoid structure with a coil shape to a quinoid structure with a linear and expanded coil shape. Second, the composition ratio changes on the film surface as the number of conductive PEDOT molecules increases and the number of insulating PSS molecules decreases. Third, the size of the PEDOT:PSS polymer aggregation decreases and becomes uniformly distributed on the surface, thereby improving the network with neighboring particles. In conclusion, these changes improve the electrical conductivity of the PEDOT:PSS film.

Finally, the Salisbury screen absorber was designed, manufactured, and evaluated in order to study the applicability and performance of the developed PEDOT:PSS–polyurethane polymer-based conductive paste. This absorber was manufactured through the same process with the actual radar-absorbing structure, and the performance was also evaluated using free space measurement equipment. According to the experimental results, the fabricated Salisbury screen absorber resonated at 9.7 GHz, the reflection loss was −38.6 dB, and the 90% absorption bandwidth was 3.4 GHz (8.2 to 11.6 GHz), meaning that it showed excellent performance. Therefore, the applicability of the synthesized PEDOT:PSS-based conductive paste was sufficiently verified and it is expected that this paste will be able to exhibit excellent performance when applied to the screen-type radar absorbers.

## Figures and Tables

**Table 1:** Materials used in the conductive paste.

Category	Product Name	Characteristics
Conductive Material	Clevios PH 500	- High stability/pH 1.5–2.5 - Max. conductivity: 300 S/cm - Viscosity: 8–25 mPa∙s
Binding Material	NPC-3600	- Chemical and thermal stability - pH 8.5/Viscosity: 1000 mPa∙s - Hardness: 200–300 Kgf/cm^2^
Secondary Compound	DMAE (Dimethylaminoethanol)	- pH control agent
	Silquest A-187	- Coupling agent
	Dynol 604	- Surface active agent
	DMSO (Dimethylsulfoxide)	- Conductive enhancer
	Tafigel PUR 40	- Thickening agent

**Table 2:** Area ratio of various components of the C(1s) signal.

Polymer	0 wt% DMSO		3 wt% DMSO		5 wt% DMSO		9 wt% DMSO	
	Binding Energy	Area Ratio	Binding Energy	Area Ratio	Binding Energy	Area Ratio	Binding Energy	Area Ratio
PEDOT/PSS	284.3 eV	47.7%	284.3 eV	52.0%	284.5 eV	52.3%	284.4 eV	51.4%
PSS	285.9 eV	47.0%	285.9 eV	42.0%	286.1 eV	41.4%	286.0 eV	41.5%
PEDOT	288.6 eV	5.3%	288.6 eV	6.0%	288.7 eV	6.3%	288.6 eV	7.1%

**Table 3:** Area ratio of various components of the S(2p) signal.

Polymer	0 wt% DMSO		3 wt% DMSO		5 wt% DMSO		9 wt% DMSO	
	Binding Energy	Area Ratio	Binding Energy	Area Ratio	Binding Energy	Area Ratio	Binding Energy	Area Ratio
PEDOT	163.7 eV	12.8%	163.8 eV	14.8%	163.8 eV	15.2%	163.9 eV	13.9%
PEDOT	164.9 eV	13.0%	165.0 eV	11.6%	165.0 eV	14.1%	165.0 eV	17.8%
PSS-Na	167.4 eV	42.0%	167.3 eV	42.6%	167.6 eV	37.7%	167.5 eV	42.4%
PSS-H	168.5 eV	32.2%	168.5 eV	31.0%	168.5 eV	33.0%	168.7 eV	25.9%

**Table 4:** Design parameters and values of the Salisbury screen absorber.

Parameter	Value	Parameter	Value
Conductivity (C_R_)	1000 S/m	Permittivity (ε′/ε″)	4.57/0.05
Substrate Size (L_S_)	161 mm	Substrate Thickness (H_S_)	3.5 mm
Sheet Thickness (H_R_)	2.65 µm	Ground Thickness (H_G_)	0.2 mm

## Data Availability

The data presented in this study are available upon request from the corresponding author.
